# Oral Health Behavior of Parents as a Predictor of Oral Health Status of Their Children

**DOI:** 10.1155/2013/741783

**Published:** 2013-05-08

**Authors:** Elham Bozorgmehr, Abolghasem Hajizamani, Tayebeh Malek Mohammadi

**Affiliations:** ^1^Oral and Dental Disease Research Center and Dental Public Health Department, School of Dentistry, Kerman University of Medical Sciences, Kerman, Iran; ^2^Research Center of Social Determinants of Health, Institute of Future Health Research, Dental Public Health Department, School of Dentistry, Kerman University of Medical Sciences, “P.O. Box 76175”, Kerman, Iran

## Abstract

*Introduction*. It is widely acknowledged that the behavior of parents affects their children's health. This study aimed to evaluate the relationship between oral health behavior of parents and oral health status and behavior of their children in a sample of preschool children in Iran. *Method and Material*. A random sample of over-five-year-old preschool children and their parents were enrolled in the study. Selection of schools was by clustering method. Parents were asked to fill a piloted questionnaire which included demographic characteristics, socioeconomic status, oral health behaviors of children and their parents. Oral health status of children was examined. The parent and their children oral health relationship were tested using regression and correlation analysis. *Results*. About 222 parents and children participated in the study. There was a significant relationship between history of having dental problems in parents and dmft index in their children (*P* = 0.01). There was a significant relationship between parental frequency of tooth brushing and child frequency of tooth brushing (*P* = 0.05); however, there was no significant relationship between parental frequency of dental visits and those of their children (*P* = 0.1). *Conclusion*. The study concluded that some important health behaviors in parents, such as tooth brushing habits are important determinants of these behaviors in their young children. So promoting parent knowledge and attitude could affect their children oral health behavior and status.

## 1. Introduction

It is widely acknowledged that the behavior of parents, and in particular mothers, affects their children's health [1]. It is also about oral health that the role of parents is very important, because they are the main caregivers of oral health to their children during the first three years of life, even in preschool, parents are still the main supplier of children's oral health [2].

Some factors such as maternal education, occupation, age, current knowledge, attitude, and behavior can provide insight for improving their health habits and their children's health indirectly [3].

The relationship between dental health of mothers and dental caries in their children can be explained by the influence of faulty dietary and hygiene habits on infants as well as by infection of the child's mouth by maternal bacteria [4, 5]. Therefore, tooth brushing habits of mothers, dietary habits, and food choices are directly associated with those of their children [5, 6]. Dental care professionals accept that efforts aimed at improving parental oral health behaviors could result in reductions in caries risk among their children [7]. However, there is limited information to confirm this relationship [6]. Furthermore, the evidence in the literature is nearly nonexistent in the context of quantifying the expected association between oral health behavior of parents and oral health of their children [6].

Some studies have examined the association between caregivers' characteristics and their children's oral health. These included caregivers' oral health behaviors and demographic factors [1, 6–12]. However, cultural norms about child-rearing differ among countries, and also they may have very different dental health care delivery systems for children that could influence these relationships. Consequently, the aim of this study was to predict any relationship between the oral health behavior of parents and oral health status and behavior of their children by using data from a sample of preschool children in Kerman, South East of Iran.

## 2. Method and Material

This report is part of an interventional study to comprise the effectiveness of oral health education using motivational interviewing comparing with traditional education on oral health of preschool children in Iran. 

A random sample of over-five-year-old preschool children and their parents were enrolled in the study. Selection of schools was by a clustering method from the list of 160 elementary schools in Kerman, and 22 children randomly enrolled from each school with their parents into the study. The volunteer parents signed an informed consent form, and the study was approved by the local research ethic committee of Kerman University of Medical Sciences. Children and their parents with systemic diseases were excluded and replaced with other children and parents. 

Only one of parents who had a more decision-making authority about eating, oral health caring, and taking a nominated child to a physician or a dentist was asked to fill a piloted questionnaire (Cronbach's alpha = 0.71).

The questionnaire included some demographic characteristics, socioeconomic statuses and oral health behaviors of children and their parents. 

Demographic characteristics and oral health behavior of children were asked from their parents. 

Oral health status of children was assessed through the following clinical examination.

1. Plaque Index (PI) using the standard plaque index (Loe and Sillnes) [13].
2. Gingival health assessment using the standard criteria modified gingival index (MGI) [14].
3. dmft index: an estimation of children's dental status by calculating the decayed, missed, and filled teeth (dmft) using the International Caries Detection and Assessment System (ICDAS) [15].

Data were analyzed by Minitab statistical software version 16. Regression analysis was used to assess the relationship between dmft of children with some demographic variables and also parental oral health behaviors. Ordinal logistic regression was used to assess the other dental health status variables (PI, GI) versus demographic characteristics and parental oral health behaviors. Correlation analysis evaluated the relationship between oral health behavior variables of parents and those of their children.

## 3. Results

From 222 children who participated in the study, 45.7 were boys, and 54.3 were girls. The mean age of parents was 33 years, ranged from 23 years to 45 years.

Eighty-nine percent of respondents were the children's mothers, 9% were the children's fathers, and 1.3% was a care giver or grandparents. One hundred and sixty-three parents (73.4%) have experienced mouth disorders during the six months before the study.  Table 1 shows oral health indices measurements. The mean dmft was 4.87, 22.1% of children had no sign of gingivitis, but severe gingivitis was observed in about 3.6% of them. The code of plaque on some index teeth surfaces was recorded as 0 in 1.8% of participants, so their dental hygiene described as excellent and 54.5% of them had a poor described oral hygiene.

Adjusted regression analysis of dmft versus demographic and behavioral variables of parents showed that there was a significant relationship between the history of having dental problems in parents and dmft index in their children (*P* = 0.01). The dmft index was not significantly related to the other variables measured in parents including parental education, frequency of tooth brushing, frequency consumption of sweet foods, and dental visits. (Table 2). 

Ordinal Logistic regression analysis showed a significant relationship between plaque index of children with mothers education (*P* = 0.00) and the history of dental problems in parents (*P* = 0.05). Plaque index of children was not significantly related to the other variables shown in Table 3.

Gingival index of children was not significantly related to other variables which were measured in parents and children (Table 3).

Correlation analysis showed a significant relationship between parental frequency of tooth brushing and child frequency of tooth brushing (*P* = 0.05). Parental frequency of consumption of sweet foods between meals had a significant relationship with this behavior in their children (*P* = 0.00), but there was no relationship between parental frequency of dental visits and those of their children (*P* = 0.1) (Table 4).

## 4. Discussion

Oral health has an important role in the general well-being of individuals. Since oral health behaviors can affect the oral health, attempting to construct good oral health behaviors can affect the general health of individuals. Indeed, the adoption of good oral health habits in childhood often takes place with parents, especially with mothers [16]. 

Since parents are the primary social force influencing child development in the early childhood years, it seems that interventions targeting parental oral health beliefs and practices may be beneficial in the prevention of oral health problems such as dental caries [17].

This study assessed some characteristics and behaviors of parents that may affect oral health behaviors and oral health statuses of their children. There was a significant relationship between the history of having dental problems in parents and dmft index in their children. Several factors, such as neglecting oral health by parents and their inability to pay for health services and genetic factors, might interpret this relationship. Parental history of dental problems may show their consideration to oral health behaviors. A lot of factors may cause poor oral health status of parents and these factors may cause poor dental health (high dmft) in children. Some previous studies have also addressed this issue [6, 18].

The relationship between plaque index of children with mother's education and history of dental problems in parents was significant. This means that the presence of dental problems in parents may follow by some problems such as poor hygiene (high-plaque index) in their children. It can also show that high dental problems in parents may affect their consideration to hygienic behaviors of their children; so, healthy parents are more likely to have children with lower plaque index than unhealthy parents. This finding confirms the previous study findings [17].

A significant relationship was found between education of mothers and plaque index of children. It can interpreted that education of mothers can increase their knowledge about health behavior followed by increasing their ability to supervise hygienic practices of their children. 

This confirms the previous studies results which have reported that parents with higher education have more positive attitudes and stronger intentions to control children's health behavior than low-educated parents [17]. In a study by Abiola Adeniyi et al., a significant relationship was reported between mothers' educational level and the oral hygiene status of their children [16].

The study showed that frequency of tooth brushing in parents is significantly in relationship with frequency of tooth brushing in their children. Tooth brushing skills and appropriateness of oral hygiene in parents can affect the frequency and quality of tooth brushing in children, because children learn many of behaviors from their parents; so, it is predictable that they follow their parents' behavior for tooth brushing.

A study by Vanagas et al. has reported that oral hygiene skills and attitudes of parents toward children oral health are significantly associated with the development of oral hygiene skills including tooth brushing in their children [19]. One study by Dye et al. in 2011 showed a direct association between tooth brushing habits of the mother and her child [6].

Parental frequency consumption of sweet foods between meals had a significant relationship with this behavior in their children. Some studies revealed a parental and child's eating behavioral relationship [20]. The results from a study by Brown and Ogden (2004) indicated that parents own eating behavior is the most important source of information for their children eating behavior [21].

There was not a significant relationship between parental frequency of dental visits and child's dental visits (*P* = 0.1). Several problems may cause parents to not take their children to a dentist. For example, large number of them may not consider primary teeth as important, while some of them visit a dentist because of their own dental problems. On the other hand, some may not consider their own teeth because of costs while their children dental health may be important for them, but some studies have found a significant relationship on this issue [6].

However, results of the study can provide an overview of the relationship between some parental factors and health and the status of their children, but generalization of them is limited because the study is cross-sectional, and the sample size is not very large.

There are also some problems in studies by questionnaires, for example, despite the emphasis on confidentiality of the project data, parental bias in responding to questions might occur. Forgetting some information by respondents is another problem of these types of studies. 

## 5. Conclusion

It can be concluded from the study results that some important health behaviors in parents, such as tooth brushing habits and frequency of consumption of sweet foods, are important determinants of these behaviors in their young children. Children with high-educated mothers had lower plaque index than the others. So, promoting parents' knowledge and attitude could affect their children oral health behavior and status.

## Figures and Tables

**Table 1:** Oral health indexes of children.

Oral health indexes of children	Frequency	Percent
Gingival inflammation index		
Sound (0)	49	22.1
Moderate (0/1–1/0)	101	45.5
Medium (1/1–2/0)	64	28.8
Severe (2/1–3/0)	8	3.6
Total	**222**	**100.0**

Plaque index		
Excellent (0)	4	1.8
Good (0/1–0/9)	25	11.3
Medium (1–1/9)	72	32.4
Poor (2-3)	121	54.5
Total	**222**	**100.0**

**Table 2:** Relationship between sociodemographic and behavioral characteristics of parents and children and dmft of their children.

Dependent variables	Independent variables	*P* value	Coefficient
	Education of fathers	0.74	0.08
	Education of mothers	0.68	0.12
dmft of children	Parental frequency of tooth brushing	0.48	−0.25
	Parental consumption of sweet foods	0.49	0.14
	Parental dental visits in the last year	0.99	0.00
	Parental history of dental problems	0.01*	−1.6

*significant.

**Table 3:** Relationship between characteristics of parents and children with oral health status of children.

Dependent variables in children	Independent variables in parents	*P* value	Odds ratio	95% CI	
				Lower limit	Upper limit
Plaque index (PI) in children	Education of fathers	0.25	0.86	0.67	1.11
	Education of mothers	0.00*	1.55	1.18	2.04
	Parental frequency of tooth brushing	0.49	0.89	0.64	1.24
	Parental consumption of sweet foods	0.20	0.88	0.72	1.07
	Parental dental visits	0.62	1.04	0.90	1.19
	Parental history of dental problems	0.05*	1.77	0.96	3.27

Gingival index (GI) in children	Education of fathers	0.69	0.95	0.75	1.21
	Education of mothers	0.32	1.14	0.88	1.48
	Parental frequency of tooth brushing	0.76	1.05	0.77	1.43
	Parental consumption of sweet foods	0.31	0.91	0.76	1.09
	Parental dental visits	0.49	1.05	0.92	1.20
	Parental history of dental problems	0.83	1.06	0.59	1.91

*significant.

**Table 4:** Relationship between oral health behaviors of parents and their children.

Health behaviors of parents	Health behaviors of children	*P* value	Pearson correlation
Frequency of tooth brushing	Frequency of tooth brushing	0.05*	0.126
Frequency of consumption of sweet foods	Frequency consumption of sweet foods	0.00*	0.28
Frequency of dental visits	Frequency of dental visits	0.1	0.105

*significant.
